# New in Town—An internet-based self-efficacy intervention for internal migrants: A randomized controlled trial

**DOI:** 10.1371/journal.pone.0299638

**Published:** 2024-03-07

**Authors:** Anna Maj, Maria Matynia, Natalia Michalak, Aleksandra Bis, Gerhard Andersson

**Affiliations:** 1 Faculty of Psychology, SWPS University, Warsaw, Poland; 2 Department of Behavioural Sciences and Learning, Department of Biomedical and Clinical Sciences, Linköping University, Linköping, Sweden; 3 Department of Clinical Neuroscience, Karolinska Institutet, Stockholm, Sweden; The Hong Kong Polytechnic University, HONG KONG

## Abstract

**Objective:**

Migration is a profound life transition that may threaten migrants’ well-being and mental health. Results of several studies suggest that social self-efficacy beliefs may be beneficial for the psychological adjustment of migrants, buffering the effect of specific stressors related to migration, helping them reduce anxiety levels, and providing support in forming of new social bonds and better integration with a new community or culture. The primary purpose of this randomized controlled trial was to examine the effectiveness of the *New in Town* internet-based self-efficacy intervention for internal migrants in Poland.

**Methods:**

Participants were 158 internal adult migrants who had changed residence in the last 6 months. They were randomized into two groups: an experimental group (receiving an internet-based self-efficacy intervention), and a waiting list control group. We examined if the intervention was effective in enhancing participants’ social self-efficacy (primary outcome), general self-efficacy, social support, satisfaction with life, and reduced reported loneliness (secondary outcomes). Outcome measures were assessed at baseline (Time 1) and 3-weeks later (Time 2). The dropout rate was 50.6%. Initially, we planned to gather follow-up data also 8-weeks after baseline (Time 3). However, due to health and safety reasons related to the COVID-19 pandemic, we decided to stop the trial. Finally, we included in our analysis only data gathered before the COVID-19 pandemic at Time 1 and Time 2.

**Results:**

A total of 159 individuals who met the study’s inclusion criteria and completed the baseline assessment were randomly assigned to either the experimental group (*n* = 80) or the waiting list control group (*n* = 79). Nevertheless, one participant assigned to the control group was excluded from the analyses because they withdrew their consent to participate after being randomized. The study results suggest that compared to the waitlist control group (*n* = 78), participants in the experimental group (*n* = 80) reported a higher level of general self-efficacy beliefs at Time 2 (Cohen’s *d* = 0.47; 95% CI: 0.15–0.79). However, there were no statistically significant effects on social self-efficacy, social support, satisfaction with life, and loneliness.

**Conclusion:**

The study offers preliminary support for the effectiveness of an internet-based self-efficacy intervention designed for internal migrants on general self-efficacy beliefs.

**Trial registration:**

The trial was registered with ClinicalTrials.gov (identifier: NCT04088487) on 11^th^ September 2019.

## Introduction

Current global estimates for the year 2019 reported around 272 million international migrants in the world, which amounts to 3.5% of the world’s population [1]. The number of international migrants increased by over 105 million (69%) between 1990 and 2017. Reports suggest that the overall number of migrants worldwide has continued to grow rapidly in recent years [2]. Migration can be defined as “the process of going from one country, region or place of residence to settle in another” (p. 2) [3]. Most of the cases involve internal movements of populations within a country, for instance from a rural district to a larger city [4]. This phenomenon is referred to as internal migration. King and Skeldon predicted that the internal mobility of households in China alone might soon exceed the present level of global international migration figures. The authors argued that the “age of migration is therefore an age of mass internal migration” (p. 1621) [4]. It is also forecasted that, by the year 2050, 140 million people could be migrating internally within their countries’ borders due to climate change [5].

Migration, as a process, involves certain phases having to do with leaving and adapting to a new culture, and with long-term separation from family and friends. This may be a source of distress for the migrants and potentially impact their mental well-being. Migration is also a risk factor of mental illnesses. This effect is due to numerous barriers that the migrating population experience in the process of migration and during the post-migration phase [3]. The list of potential stressors posing a threat to the migrant group includes, among others: communication difficulties, cultural differences, and socioeconomic as well as employment status change [6]. Migrant adolescents in Israel report worsening mental health symptoms and a high proclivity to risk behaviours compared with their native Israel counterparts [7]. Research also suggests that, in comparison to the general population, depression symptoms are more prevalent among internal migrants in China [8]. The complex relationship between health and migration is manifold. It is important to understand the “healthy immigrant” and “salmon bias” hypotheses [9,10]. The “healthy immigrant” phenomenon depicts “the observation that new migrants are healthier than their host society counterparts” (p. 1) [11]. In this case migrants—in relation to their general population of origin society—exemplify a positively selected group of people in terms of health [12,13]. Thus standing out health-wise in comparison to the destination’s country general population. However, this can be a temporary phenomenon. The advantage eventually diminishes with increased duration in the host society [14,15]. Self-selection also affects the existing pool of migrants in another way: those migrants who are in poor health are more likely to return to their societies of origin. These processes seem to be based mainly on physical, not mental health [11]. The second hypothesis, the “salmon bias effect”, describes a need to die in one’s birthplace. The hypothesis states, that numerous immigrants return to their country of origin when they expect to die [16] distorting the immigrant mortality rate.

Nonetheless, numerous factors, including age, gender, educational level, occupational background, socioeconomic context, availability of social support, and self-efficacy may impact the psychological adaptation of migrants [17,18]. Here we focus on self-efficacy as internet interventions can effectively enhance this personal resource [19]. Moreover, self-efficacy was highlighted as one of the significant factors that help an individual cope with stressful life transitions and adjust to a new situation [20]. Jerusalem and Mittag suggested that “within this stressful transitional adaptation to the new societal living conditions, self-efficacy can function as a personal resource protecting against deleterious experiences, negative emotions, and health impairment” (p. 179) [20].

A basic distinction can be made between general and domain-specific self-efficacy. The Social Cognitive Theory defines general self-efficacy as “belief in one’s capabilities to organize and execute the course of action required to produce given attainments’’ (p. 3) [21]. Narrowing the self-efficacy construct scope in the course of research to a chosen domain or context has substantial advantages, as using domain-specific self-efficacy measures leads to a better prediction of the outcomes [22]. The present study investigated the efficacy of New in Town, an internet intervention focused on the enhancement of internal migrants’ social self-efficacy. Social self-efficacy is defined as "confidence in one’s ability to engage in the social interactional tasks necessary to initiate and maintain interpersonal relationships in social life and career activities” (p. 98) [23]. Therefore, social self-efficacy is an invaluable personal resource when it comes to facing challenges related to migration, such as building a new social network. Studies targeting first-year migrant students have revealed a number of benefits of high social self-efficacy for mental health-related outcomes. A recent study investigating the relationship between social-self efficacy and loneliness among Chinese international students in the United States indicated a bidirectional relation between these factors [24]). Moreover, individuals with high social self-efficacy reported having more American friends in their social circle [24]. In line with these results, research conducted among internal migrants in the USA—first-year college students, has shown social self-efficacy to negatively correlate with loneliness, depression, and anxiety [25]. Another study targeting international students in the United States as a population revealed that high social self-efficacy was negatively related to acculturative stress, depression, and self-concealment [26]. On the other hand, low social self-efficacy predicted low academic satisfaction and high distress among international students in Canada [27] and Australia [28]. The studies mentioned above pointed out social self-efficacy as a crucial factor for migrants’ psychological well-being.

Self-efficacy beliefs can be effectively enhanced using internet-based interventions—"computerised programs or service delivered through the Internet (e.g. a website), designed to create a positive change in behaviour or health" (p. 2) [29]. To the best of our knowledge, there is no prior research on internet interventions targeting migrants’ social-self efficacy. Extensive research suggests that internet interventions can lead to participants’ self-efficacy reinforcement in other domains: work [30], healthy eating [31], reduced cannabis use [32], and even trauma distress coping [33]. Furthermore, another internet intervention designed on the same theoretical framework as the New in Town intervention (namely, the Social Cognitive Theory) was found effective for enhancing trauma-coping self-efficacy [19]. Human service professionals exposed to indirect trauma displayed significantly greater self-efficacy beliefs related to managing secondary traumatic stress compared to respondents in an active control group. Given the many benefits offered by the use of internet interventions, including increased accessibility, cost-effectiveness, flexibility, and increased patient engagement in the therapy process [34], we decided to design our own program to offer dedicated support for internal migrants.

As mentioned above, the New in Town intervention is based on the Social Cognitive Theory by Bandura, identifying four sources of self-efficacy beliefs. The first is *mastery experience* rooted in the perception of connections between one’s actions and consequences. The second is the *vicarious experience* (modeling) which relies on observation of behaviours of others (models)—and not only for the effects of their actions, but also for recognition of strategies of actions that led to the given effects. The third source of self-efficacy beliefs is persuasion that an individual has the necessary abilities to perform tasks. It comes through *verbal and social persuasion* used to encourage feedback about one’s abilities to succeed. *Psychological and emotional states* constitute the fourth source. Negative emotions and stress experienced by individuals may alter their perception and evaluation of their own capabilities. For example, anxiety leads to a lower sense of self-efficacy. However, a positive psychological state can make it easier for individuals to recall their successes [35]. The intervention aims to enhance social self-efficacy through interactive exercises based on Cognitive Behavioural Therapy (CBT) techniques. The exercises are structured in eight modules and relate to the sources of self-efficacy beliefs identified by Bandura.

The present trial aimed to examine the New in Town internet intervention’s effects on Polish adult internal migrants in a two-armed randomized controlled trial. Migration can be defined as “movements of the population connected with changing the place of residence (permanent residence or temporary stay) involving crossing the border of the administrative unit of the territorial division of Poland (internal migration) or the national border (international migration)” (p. 33) [36]. The trial was conducted in 2020, before the war in Ukraine, which changed the demographic situation in Poland [37]. Before Russia’s invasion of Ukraine, in the general Polish population, internal migration was the most common and international immigration rarer. In fact, the inflow of migrants in 2019 in Poland consisted of 96.52% internal migrants and only 3.48% international migrants [38]. It is also worth noting that, compared to northern and western European countries, such as Norway, the Netherlands and the UK, the mobility in Poland before the war in Ukraine was quite low [39]. When it comes to internal migration intensity, Poland was similar to Estonia, the Czech Republic, and Romania. Rees and Kupiszewski suggest that these differences between European countries in migration intensity “seem to be associated with level per capita incomes (low incomes equal low mobility) and with the degree to which housing is supplied by the state sector (the barriers to migration within state sector housing are considerable) (p. 34) [39]. Results of a more recent study confirmed that the mobility in Poland in the first two decades of the 21st century was one of the lowest in Europe, with the average number of moves in early and mid-adulthood being 1.80, compared to 5.01 in Denmark and 4.61 in the UK [40].

To be easily accessed by internal migrants, the intervention was provided in the Polish language. We selected participants from those reporting to have changed their residence place in the last six months. They were then randomized into two groups: a self-help internet-based intervention or a waiting list control group. We tested whether the intervention would have a positive effect in enhancing migrants’ social self-efficacy (primary outcome), general self-efficacy, social support, satisfaction with life, and reduced reported loneliness (secondary outcomes). This is a deviation from the study protocol, as we did not initially include general self-efficacy as a secondary outcome. Outcome measures were assessed at baseline (Time 1) and 3-weeks later (Time 2). Initially, we also planned to gather follow-up data also 8-weeks after baseline (Time 3). Ultimately, we decided to limit our analysis to data gathered before the COVID-19 pandemic [41] at Time 1 and Time 2 measurement points. Following the Research Ethics Committee of the Faculty of Psychology at the SWPS University in Warsaw guidelines, the study participants access to the interventions was blocked on 16t^h^ March 2020. Due to health and safety reasons, it was necessary as the intervention content was designed to encourage face-to-face social interactions. For this reason, data could not be gathered at the Time 3 point, contrary to the original plan.

## Methods

### Trial design

The New in Town study was a two-armed randomized controlled trial in a parallel design comparing post-test effects in an experimental (the New in Town intervention) and control condition (waitlist control group) (see the CONSORT Checklist, S1 File).

The study was approved on 15th January 2019 by the Ethics Committee of the Faculty of Psychology at the SWPS University in Warsaw, Poland (Approval ref. No. 4/2019, Appendix 1 approval ref. No. 30/2019, Appendix 2 approval ref. No. 46/2019), and was registered at ClinicalTrials.gov (identifier: NCT04088487) on 11th September 2019. The study protocol has been published [42].

### Participants and recruitment

Participants were recruited through social and traditional media campaigns targeted mainly at first-year university students in Poland. The recruitment lasted from 14^th^ January to 16^th^ March 2020. The New in Town intervention presented content encouraging people to engage in social interactions but was not adapted for the COVID-19 pandemic situation [41]. Therefore, the recruitment was suspended following the recommendations of the Ethics Committee of the Faculty of Psychology at the SWPS University in Warsaw (16^th^ March 2020). The inclusion criteria were as follows: 1) at least 18 years of age, 2) having changed the place of residence in the last 6 months. The exclusion criteria were the lack of Internet access and no knowledge of the Polish language.

### Power calculation

Initially, we planned to recruit 100 participants (see the final Trial study protocol accepted by the Ethics Committee, S2 and S3 Files). However, following the suggestions of the study protocol reviewers [42], we conducted a power analysis that resulted in a sample size of 182 participants. Although, taking into account a deviation from the study protocol, as we did not initially include general self-efficacy as a secondary outcome, the analyses in our study encompass five, not four outcomes. Therefore, we applied Bonferroni’s adjustment to address multiple comparisons which lowered the probability level to 0.01, not 0.0125. Additionally, results of previous studies suggest a medium effect of internet-based interventions aiming to enhance self-efficacy beliefs [43,44]. Considering an anticipated moderate effect size (*d* = 0.50), an adjusted probability level of 0.01, and a statistical power of 0.80, the power analysis conducted using G*Power [45] determined a required sample size of 191 participants.

### Intervention

The New in Town self-help intervention is based on a cognitive-behavioural approach and consists of eight modules (see Table 1).

**Table 1:** The content of the New in Town intervention.

Modules	Aims	Exercises
Introduction	▪ To provide basic psychoeducation on self-efficacy	-
Our successes	▪ To provide psychoeducation on sources of self-efficacy ▪ To increase the frequency of exposure to social situations ▪ To enhance self-efficacy through mastery experience	Giving Compliment Exercise
Negative thoughts	▪ To provide psychoeducation on the role of negative thoughts on emotions, somatic experience, and behaviour ▪ To provide cognitive techniques	Cognitive Restructuring Exercise
Social models	▪ To increase the frequency of exposure to social situations ▪ To enhance self-efficacy through vicarious experiences	Social Model Exercise
Hobby	▪ To encourage engaging in enjoyable leisure activities ▪ To present the benefits of outdoor activities	Outdoor Activities Search
You can do it!	▪ To provide psychoeducation on the role of social support ▪ To increase the frequency of exposure to social situations ▪ To enhance self-efficacy through social persuasions ▪ To create a social support network	Buddy support exercise
A sound mind in a sound body	▪ To provide psychoeducation on emotional and physiological states as a source of self-efficacy ▪ To enhance awareness of own needs ▪ To practice relaxation skills	Self-care exercise
Problem-solving	▪ To provide psychoeducation on goal setting ▪ To practice interpersonal skills	Goal setting exercise

Users are asked to complete modules in sequential order. Completed modules are not blocked, and users may revisit them at will. There are no time limits. Each module contains psychoeducation, and seven out of the eight modules are accompanied by an exercise. Some of the exercises require social interactions.

The first module provides insight into the concept of self-efficacy, and it explains how self-efficacy beliefs may have an impact on adaptation to the new place of residence. The next modules present four sources of self-efficacy: 1) mastery experiences, 2) vicarious experiences, 3) verbal persuasions, and 4) emotional and physiological states [21]. Users are provided with cognitive restructuring tools to deal with negative thoughts, a case study of a person trying to establish new relationships, and tasks that aim to exercise social skills. In addition, users are taught awareness of their own emotional and physiological states and relaxation skills. The intervention requires participation in outdoor activities and social skills training. Exercises are evidence-based and dedicated to internal migrants. The intervention is available only in Polish.

### Procedure

Applications for study participation were registered via the study website. The authors had access to the e-mail addresses of study participants during and after data collection. The respondents were encouraged to set up new e-mail accounts dedicated solely to the purpose of participating in the study. After completion of the screening, participants were automatically redirected to the baseline assessment (Time 1). Only those respondents who met all inclusion criteria and signed a web-based informed consent were randomized by an independent researcher and assigned with a 1:1 ratio between the experimental group and the waitlist control group. To ensure an equal distribution of participants across both study conditions, we employed non-stratified block randomization, with two participants allocated per block. A predefined randomization protocol was generated using randomizing software (randomizer.org). Information about group allocation was sent within 2 days from baseline. Participants assigned to the experimental group completed the baseline assessment between January 14th and March 4th, 2020, and were provided with New in Town login details immediately after group allocations. For the waitlist control group, who completed the baseline between January 14th and March 13^th^, 2020, the intervention was provided 8 weeks after the assessment. Both groups received intervention access for 3 weeks and were asked to complete modules at their own pace. The post-test assessment (Time 2) for both groups was conducted 3 weeks after the baseline.

**Fig 1:**
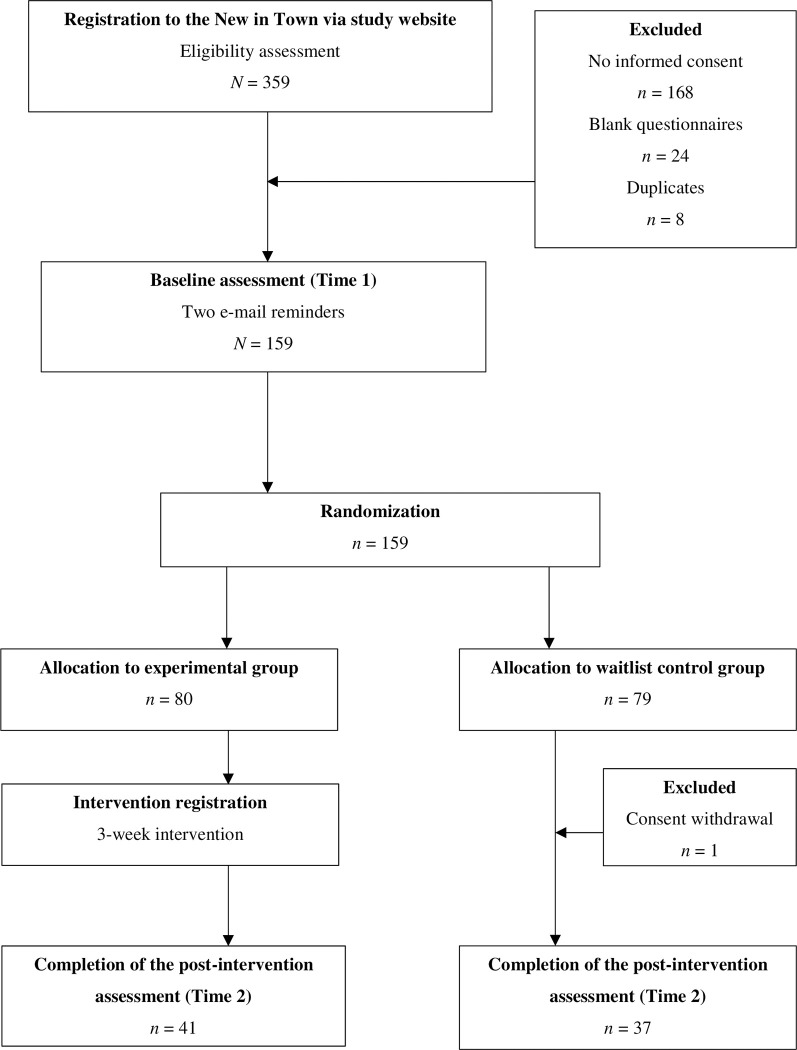
Flow of participants.

All assessments were provided online. Participants were asked via e-mail to complete the post-test assessment. The e-mail reminders were sent twice if the questionnaire was not completed in one week. All data, including informed consent, was stored in a cloud-based environment (SurveyMonkey). Fig 1 presents the study flow chart.

### Measurements

The descriptive statistics for primary and secondary outcomes measures and between-group effect sizes at post-test (Cohen’s *d* based on adjusted means and pooled adjusted standard deviations) are presented in Table 2.

**Table 2:** Means, standard deviations and between group effect sizes (Cohen’s *d*).

Variable	Condition	*M* (*SD*)		Cohen’s *d* (95% Cl)
		T1	T2	
General self-efficacy	Experimental group	3.31 (0.69)	3.35 (0.68)	0.47 (0.15, 0.79)
	Waitlist control group	3.11 (0.77)	3.00 (0.74)	
	Total	3.21 (0.73)	3.18 (0.73)	
Social self-efficacy	Experimental group	3.06 (0.88)	3.15 (0.78)	-0.08 (-0.39, 0.23)
	Waitlist control group	2.76 (0.88)	2.96 (0.75)	
	Total	2.91 (0.89)	3.06 (0.77)	
Loneliness	Experimental group	2.99 (0.82)	2.87 (0.80)	-0.29 (-0.61, 0.02)
	Waitlist control group	3.06 (0.93)	3.07 (0.85)	
	Total	3.03 (0.88)	2.97 (0.83)	
Perceived social support	Experimental group	3.07 (0.61)	3.13 (0.67)	0.39 (0.07, 0.70)
	Waitlist control group	2.97 (0.70)	2.88 (0.75)	
	Total	3.02 (0.66)	3.01 (0.72)	
Need for support	Experimental group	3.04 (0.65)	3.02 (0.59)	-0.29 (-0.60, 0.03)
	Waitlist control group	3.00 (0.65)	3.11 (0.55)	
	Total	3.02 (0.65)	3.06 (0.57)	
Support seeking	Experimental group	2.93 (0.64)	2.88 (0.69)	0.02 (-0.30, 0.33)
	Waitlist control group	2.81 (0.62)	2.78 (0.61)	
	Total	2.88 (0.63)	2.83 (0.65)	
Satisfaction with life	Experimental group	4.02 (1.30)	4.09 (1.23)	-0.21 (-0.52, 0.11)
	Waitlist control group	3.81 (1.39)	4.09 (1.32)	
	Total	3.92 (1.35)	4.09 (1.27)	

*M–*mean, *SD–*standard deviation, T1 –Time 1 (baseline assessment), T2 –Time 2 (post-test assessment), Cl–confidence interval. Cohen’s *d* was calculated based on adjusted means and adjusted standard deviations at post-test derived from the analysis of covariance.

#### Primary outcome measure

*Social self-efficacy* was measured using the General Self-Efficacy Scale (GSES) [46]. This measure contains two subscales: 1) generalized beliefs about self-efficacy (general self-efficacy, GSE) and 2) beliefs about self-efficacy in establishing and maintaining relationships with others (social self-efficacy, SSE). The whole scale consists of 30 items, 7 of which are buffer theorems. The SSE subscale consists of 6 items. Participants responded to the items on a 5-point Likert scale (1 = strongly disagree, 5 = strongly agree). Higher scores reflect a higher level of self-efficacy. The Cronbach alphas for the SSE subscale in the present study were .81 at Time 1 and .75 at Time 2.

#### Secondary outcome measures

*General Self-efficacy* was assessed using the General Self-Efficacy subscale of the GSES described above [46]. The GSE subscale consists of 17 items. The Cronbach alphas obtained for this subscale were high: .90 at Time 1 and .91 at Time 2.

*Loneliness* was assessed using the 11-item De Jong Gierveld Loneliness Scale [47,48]. Each item is measured on a 5-point Likert scale ranging from 1 (definitely yes) to 5 (definitely no). Positive items were reversed, and higher scores represented greater loneliness. In this study, the reliability of the scale was .90 at Time 1 and .92 at Time 2.

*Social support* was measured using the Berlin Social Support Scale (BSSS) [49]. The scale consists of 6 subscales: perceived available support, need for support, support seeking, actually received support, provided support, and protective buffering scale. In this study, we applied the three subscales: available support (8 items), need for support (4 items), and support seeking (5 items). Negative items were reversed. Higher BSSS scores reflect higher levels of perceived support and need for support. The response scale ranged from 1 (strongly disagree) to 4 (strongly agree). The Cronbach alphas obtained in this study were satisfactory, for the perceived social support scale it was .91 at Time 1 and .94 at Time 2, the need for support scale was .74 at Time 1 and .66 Time 2, and for support seeking .76 at time 1 and .79 at Time 2.

*Satisfaction with Life* was assessed using the 5-item Satisfaction with Life (SWLS) [50]. Participants were asked to respond to the items on a 7-point scale (1 = strongly disagree, 7 = strongly agree). The Cronbach alphas were high, .87 at Time 1 and .89 at Time 2.

#### Other measures

*Demographic Data Questionnaire*. Participants completed the demographic data questionnaire to collect data on gender, age, education, job seniority, the previous and present place of residence (rural area, city with up to 20,000 inhabitants, city with up to 100,000 inhabitants, city with up to 500,000 inhabitants, city with over 500,000 inhabitants). This measure was applied only at Time 1.

*User Experience Questionnaire (UEQ)* [51] is a psychometric tool based on semantic differential to assess user experience related to interaction with an interactive product. The experimental group participants filled out UEQ at the T2 measurement point to assess the usability of the online intervention. The scale consists of 26 items, each encompassing a pair of opposite adjectives (like unattractive and attractive). Users rate an intervention using the 7 points Likert scale, starting with -3 and ending on 3, where -3 is the most negative, 0 is neutral, and 3 is the most positive answer. Items create six scales: Attractiveness, Perspicuity, Efficiency, Dependability, Stimulation, and Novelty. *Attractiveness*, the most generic dimension, measures the overall impression of the product and whether the user likes it. *Perspicuity* is an indicator of how easy it is to understand and learn how to use the product. *Efficiency* assess if users perceive completing tasks using a product as effortless and straightforward. *Dependabilit*y is related to feeling in control of the interaction with the product and its predictability. *Simulation* is measuring how engaging and motivating is the use of the product. Finally, *Novelty* refers to innovation and product creativity [51].

The scale is reliable, Cronbach’s alpha coefficient for the English version of the questionnaire has the following values for specific subscales: Attractiveness .86, Perspicuity .71, Efficiency .79, Dependability .69, Stimulation .88 and Novelty .84 [52]. The reliability of the scales in the current study was respectively: Attractiveness .92, Perspicuity .81, Efficiency .72, Dependability .52, Stimulation .84, and Novelty .68.

### Statistical analysis

Baseline characteristics are presented descriptively by randomized groups, including means (*M*) and standard deviations (*SD*) for continuous variables, along with frequency counts and percentages for categorical variables. The independent samples t-tests and chi-square tests for categorical data were used to conduct dropout analysis, in which we compared study participants who dropped out (dropouts) with those who completed the study post-test assessment (completers). Pearson’s correlations were calculated to evaluate the associations among the study variables.

To verify the intervention effect on the outcomes (self-efficacy, loneliness, social support, and satisfaction with life), we completed an analysis of covariance (ANCOVA), controlling baseline levels of the respective outcome. If participants are allocated randomly to study conditions, this method of analysis has been shown to have more power than analysis of variance (ANOVA) [53,54]. We applied Bonferroni adjustment for multiple comparisons, which lowered the probability level to 0.01 [55]. The obtained results represent the short-term effect. The aforementioned analyses were conducted on the intention-to-treat sample (*N* = 158). According to the intention-to-treat concept “all patients randomly allocated to one of the treatments in a trial should be analysed together as representing that treatment, whether or not they completed, or indeed received that treatment” (p. 837) [56]. Additionally, we calculated Cohen’s *d* based on adjusted means and pooled adjusted standard deviations for the between-group differences at post-test (Time 2). Standard deviations were derived from the standard errors using the formula *SD* = SE * √N. Missing data were handled using the multiple imputation (MI) procedure with 25 iterations. The imputation procedure was performed using all baseline scores, all available post-test scores, and all observed participants’ data that missingness is related to (see *Dropout Analysis*) as predictors [57]. All analyses were conducted using IBM SPSS Statistics 26.0.

The analysis of user experience perceptions was conducted on participants assigned to the experimental group who completed the post-test assessment (Time 2) (*N* = 41).

## Results

### Sample

A total of 159 people met the inclusion criteria and completed the baseline assessment. One participant randomized to the control group was excluded from the analyses because of the withdrawal of consent for study participation after randomization. Participants were, therefore, 158 internal migrants with an average age of 25.32 years (*SD* = 6.51). Age structure ranged between 18 and 49 years. Women constituted 81.6% (*N* = 129) of the sample. The majority of the sample was either single (50%) or cohabiting (38%). They were employed for a period of anywhere between 0 to 26 years, with a mean of 4.68 years (*SD* = 5.24). Participants changed the place of residence on average 4.33 months prior to the date of report (*SD* = 1.78). Overall, most respondents (65.8%) moved to a city of up to 500,000 inhabitants. A detailed description of the sample is presented in Table 3.

**Table 3:** Baseline characteristics by randomised group.

Variable	Total *N* = 158		Experimental group *n* = 80		Waitlist control group *n* = 78	
Female gender, *n* (%)	129 (81.6)		64 (80)		65 (83.3)	
Education, *n* (%)						
Primary or lower secondary	1 (0.6)		0 (0)		1 (1.3)	
Secondary	82 (51.9)		41 (51.2)		41 (52.6)	
Higher education	69 (43.7)		37 (46.3)		32 (41)	
Above higher education	5 (3.2)		1 (1.3)		4 (5.1)	
Previous place of residence, *n* (%)						
Rural area	27 (17.1)		13 (16.3)		14 (17.9)	
City with up to 20,000 inhabitants	19 (12)		10 (12.5)		9 (11.5)	
City with up to 100,000 inhabitants	35 (22.2)		15 (18.8)		20 (25.6)	
City with up to 500,000 inhabitants	32 (20.3)		14 (17.5)		18 (23.1)	
City with over 500,000 inhabitants	45 (28.5)		28 (35)		17 (21.8)	
New place of residence, *n* (%)						
Rural area	5 (3.2)		4 (5)		1 (1.3)	
City with up to 20,000 inhabitants	3 (1.9)		2 (2.5)		1 (1.3)	
City with up to 100,000 inhabitants	14 (8.9)		8 (10)		6 (7.7)	
City with up to 500,000 inhabitants	32 (20.3)		14 (17.5)		18 (23.1)	
City with over 500,000 inhabitants	104 (65.8)		52 (65)		52 (66.7)	
Marital status, *n* (%)						
Single	79 (50)		42 (52.5)		37 (47.4)	
Cohabiting	60 (38)		29 (36.3)		31 (39.7)	
Married	13 (8.2)		6 (7.5)		7 (9)	
Divorced	5 (3.2)		2 (2.5)		3 (3.8)	
Widowed	1 (0.6)		1 (1.3)		0 (0)	
Age (years), *M* (*SD*)	25.32 (6.51)		25.23 (6.40)		25.41 (6.67)	
Duration of employment (years), *M* (*SD*)	4.68 (5.24)		4.95 (5.55)		4.40 (4.92)	

*M–*mean, *SD–*standard deviation.

### Preliminary results

#### Dropout analysis

Out of the 158 participants included in the analyses, 78 completed the post-test assessment (Time 2). The overall dropout rate, defined as a loss to post-test, was therefore 50.6%, with a 48.8% dropout rate in the experimental group and 52.6% in the waitlist control group, respectively. The Little’s test showed that data were not missing completely at random (χ^2^ = 29.11, *p* = .01).

Dropouts and completers did not differ in age; *t*(131.99) = -1.77, *p* = .08, education χ^2^ = (3, *N* = 157) = 3.00, *p* = .39, marital status χ^2^ = (4, *N* = 158) = 6.23, *p* = .18, number of months since change of place of residence *t*(155) = -1.17, *p* = .24, general social self-efficacy at Time 1 *t*(156) = 0.47, *p* = .64, social self-efficacy at Time 1 *t*(156) = 0.06, *p* = .95, loneliness at Time 1 *t*(156) = 0.67, *p* = .50, support seeking at Time 1 *t*(156) = -1.82, *p* = .07 and satisfaction with life at Time 1 *t*(156) = -1.64, *p* = .10.

Analyses revealed that completers differed significantly from dropouts from the study across four variables: gender χ^2^ = (1, *N* = 157) = 4.19, *p* = .04, duration of employment *t*(116.73) = -2.16, *p* = .03, perceived social support at Time 1 *t*(152.46) = -2.05, *p* = .04 and need for support at Time 1 *t*(152.30) = -2.34, *p* = .02. Participants who completed both assessments presented higher levels of perceived social support (*M* = 3.13, *SD* = 0.59), need for support (*M* = 3.14, *SD* = 0.58), and duration of employment (*M* = 5.65, *SD* = 6.23) than those who dropped out, with perceived social support (*M* = 2.92, *SD* = 0.70), need for support (*M* = 2.90, *SD* = 0.69), duration of employment (*M* = 3.77, *SD* = 3.93). Finally, the dropout rate for women (*N* = 60) was much higher than for men (*N* = 19). The results confirmed the missing at random (MAR) pattern, with missingness related to gender, duration of employment, perceived social support at Time 1 and need for support at Time 1.

#### Associations among the study variables

Pearson’s correlations of main study variables are reported in Table 4. Higher general and social self-efficacy were related to higher satisfaction with life and higher perceived social support. The associations were reported at both measurement points (Time 1, Time 2). Higher loneliness was associated with lower self-efficacy, satisfaction with life and perceived social support at Time 1 and Time 2. The need for support was negatively correlated with general self-efficacy at both measurement points. The support seeking at Time 1 was positively correlated with social self-efficacy at Time 1, perceived social support (Time 1 and Time 2), and need for support at (Time 1 and Time 2). On the other hand, support seeking at Time 2 was positively related to satisfaction with life (Time 1 and Time 2), perceived social support (Time 1 and Time 2), and need for support (Time 1 and Time 2).

**Table 4:** Correlations between the study variables (N = 158).

	1	2	3	4	5	6	7	8	9	10	11	12	13
1. General self-efficacy T1	-												
2. General self-efficacy T2	.83**	-											
3. Social self-efficacy T1	.51**	.47**	-										
4. Social self-efficacy T2	.55**	.45**	.83**	-									
5. Loneliness T1	-.52**	-.43**	-.48**	-.48**	-								
6. Loneliness T2	-.55**	-.58**	-.38**	-.46**	.80**	-							
7. Satisfaction with life T1	.59**	.53**	.36**	.41**	-.56**	-.60**	-						
8. Satisfaction with life T2	.49**	.43**	.26**	.37**	-.44**	-.59**	.80**	-					
9. Perceived social support T1	.38**	.27**	.28**	.38**	-.68**	-.64**	.47**	.40**	-				
10. Perceived social support T2	.46**	.45**	.30**	.33**	-.55**	-.72**	.45**	.51**	.75**	-			
11. Need for support T1	-.28**	-.25**	.04	-.02	.16*	.19*	-.13	-.03	.15	.04	-		
12. Need for support T2	-.16*	-.17*	.01	-.05	.11	.06	-.06	.05	.19*	.27**	.71**	-	
13. Support seeking T1	-.12	-.01	.19*	.15	-.03	-.05	.06	.04	.25**	.23**	.61**	.48**	-
14. Support seeking T2	.09	.06	.09	.12	.00	-.14	.17*	.22**	.24**	.37**	.51**	.62**	.68**

*p < 0.05

**p < 0.01. T1 –Time.

### Effects on self-efficacy

Analyses of covariance were conducted separately for general self-efficacy (GSE) and social self-efficacy (SSE). The analyses for GSE (with general self-efficacy at Time 1 included as the covariate) presented a significant effect of the group assignment on GSE at Time 2; *F*(1,155) = 8.68, *p* = .004, partial η^2^ = 0.05. Overall, the experimental group participants reported a higher GSE at Time 2 than waitlist control group (see Table 2). The covariate, GSE at Time 1, was significantly related to the GSE at Time 2, *F*(1,155) = 332.75, *p* < 0.001, partial η^2^ = 0.68. However, the effect of the group assignment on SSE at Time 2 (with social self-efficacy at Time 1 included as the covariate) was not significant; *F*(1,155) = 0.27, *p* = .61, η^2^ = 0.00. On the other hand, the covariate, SSE at Time 1, was significantly related to the SSE at Time 2, *F*(1,155) = 333.06, *p* < 0.001, partial η^2^ = 0.68.

### Effects on loneliness

The analysis of covariance (with loneliness at Time 1 included as the covariate) yielded a non-significant effect of a group assignment on loneliness at Time 2; *F*(1,155) = 3.41, *p* = .07, partial η^2^ = 0.02. The covariate was significantly related to the loneliness at Time 2, *F*(1,155) = 278.11, *p* < 0.001, partial η^2^ = 0.64.

### Effects on social support

Analyses of covariance were conducted separately for each of the three social support outcomes (perceived social support, need for support, support seeking). The effects of group assignment on perceived social support at Time 2 (perceived social support at Time 1 as the covariate), *F*(1,155) = 5.87, *p* = .017, partial η^2^ = 0.04, need for support at Time 2 (need for support at Time 1 as the covariate), *F*(1,155) = 3.23, *p* = .07, partial η^2^ = 0.02, and on support seeking at Time 2 (support seeking at Time 1 as the covariate), *F*(1,155) = 0.01, *p* = .92, partial η^2^ = 0.00, were not significant (Bonferroni correction for multiple tests lowered *p* levels to 0.01). The effect of covariate was significant for all three outcomes: perceived social support, *F*(1,155) = 205.14, *p* < 0.001, partial η^2^ = 0.57, need for support, *F*(1,155) = 157.98, *p* < 0.001, partial η^2^ = 0.50, and support seeking, *F*(1,155) = 131.77, *p* < 0.001, partial η^2^ = 0.46.

### Effects on satisfaction with life

Analysis of covariance (with satisfaction with life at Time 1 entered as the covariate) revealed a non-significant effect of a group assignment; *F*(1,155) = 1.67, *p* = .20, η^2^ = 0.01. The covariate was significantly related to the satisfaction with life at Time 2, *F*(1,155) = 285.35, *p* < 0.001, partial η^2^ = 0.65.

### User experience perception

When assessing user experience perceptions, we used data from completers assigned to the experimental condition (*N* = 41), so the actual intervention users. User experience of intervention users was rated as moderate (UEQ) [51] assessed above average in the dimension of Perspicuity (*M* = 1.54, *SD* = 1.44) and below average in the dimensions of Dependability (*M* = 0.99, *SD* = 0.72), Stimulation (*M* = 0.67, *SD* = 1.12), Attractiveness (*M* = 1.18, *SD* = 1.15) and Efficiency (*M* = 0.99, *SD* = 0.97) and Novelty (*M* = 0.22, *SD* = 0.72) against the benchmarks provided by Schrepp et al. [58] based on data from 468 studies (*N* = 21175 persons) targeting variety of digital products.

## Discussion

The present randomized controlled trial aimed to examine the effectiveness of the New in Town internet-based intervention for internal migrants in Poland. The results indicate that, compared to the waitlist control group, participants in the experimental group reported higher general self-efficacy beliefs at post-test (Time 2). At the same time, the effect of the intervention participation on social self-efficacy, social support, satisfaction with life, and loneliness reported levels were not statistically significant.

To the best of our knowledge, this trial is the first to examine an internet-based self-help intervention aimed at increasing social self-efficacy among internal migrants [42]. Results indicate that the New in Town intervention effectively improved general self-efficacy beliefs, but was not effective in enhancing domain-specific self-efficacy beliefs related to social interactions (primary outcome). It was also not effective in increasing social support, satisfaction with life, and loneliness (the remaining secondary outcomes). One possible explanation for these results may be that the 3-weeks interval between measurement times in our trial may be too short to observe the intervention’s effect on study outcomes. A previous study on internet intervention aimed at increasing domain-specific self-efficacy suggests that participants in the experimental group show greater self-efficacy beliefs related to managing secondary traumatic stress compared to a control group at the 1-month follow-up [19]. Initially, we planned to assess outcome measures at three time points: before the intervention (baseline, Time 1), 3 weeks after the baseline (post-test, Time 2), and 8 weeks after the baseline (follow-up, Time 3). Unfortunately, the COVID-19 pandemic interrupted data gathering [41]. The content of the New in Town intervention encouraged face-to-face social interactions. Therefore, we were forced to block participants’ access to the intervention due to health and safety reasons, and our analysis is limited to data gathered before the pandemic (Time 1 and Time 2). Consequently, the obtained results represent only the short-term effect of the New in Town intervention. There is no doubt that social self-efficacy could be built on, among others, experiences of positive interpersonal interactions in a real-world setting [23]. Due to the aforementioned and unforeseen circumstances, this essential part of the intervention was restricted to a shorter period than initially planned, and this also applies to the findings. Another explanation could be that the specific modality of the intervention’s method of delivery (internet-based, self-help with no therapist guidance) may be the reason why it has no effect on social self-efficacy and the remaining secondary outcomes (social support, satisfaction with life, and loneliness). What is more, experimental group participants reported a quite poor user experience, which could also affect study results. A possible explanation for these results may be also that the secondary outcomes mentioned above are related to other factors, not controlled in our trial, such as the socioeconomic context or received social support, that may impact the functioning and well-being of study participants during this stressful life transition [17,18].

Moreover, it is possible that the content of the intervention is too general and does not explicitly address social self-efficacy beliefs, but refers to the general ones. Exercises contained in the intervention relate to the four sources of self-efficacy beliefs mentioned in the Social Cognitive Theory: mastery experiences, vicarious experiences, verbal persuasions, and emotional and physiological states [35]. Therefore, the content of the New in Town is theory-driven, and the intervention itself proved to be effective in increasing general self-efficacy. It is worth noting that not only social self-efficacy beliefs but also the general self-efficacy ones may have positive effects on migrants’ well-being. Research has shown that migrants with high levels of general self-efficacy report better health, subjective well-being, interaction and work adjustment, and lower anxiety and psychological distress, compared to migrants with low level scores [20,59,60]. Therefore, migrants may still benefit from participation in the New in Town programme through general self-efficacy enhancement. Providing effective and theory-driven internet intervention targeting migrants’ well-being is especially important in light of the fact that more than 30% of Polish citizens are internal migrants [61]. Implementation of an easily accessible intervention to target this segment of the population may be a way of responding to the needs of millions of potential users. The results are therefore promising and require further investigation.

Finally, it is also possible that participants were less eager to engage in exercises that require social interactions, for reasons dictated by the COVID-19 outbreak [41]. Although we decided to stop the trial on 16^th^ March 2020, four days after World Health Organization [62]) announced the COVID-19 outbreak as a pandemic, Polish media had widely spread the news about SARS-CoV-2 since February 2020 [63]. Along with exercises that involve interactions with other people, The New in Town intervention includes, among other things, psychoeducation on self-efficacy and its sources, exercises aimed to build participants’ awareness of their own emotional and physiological states, and support in dealing with negative thoughts. We did not verify whether the participants had managed to complete their assignments that required face-to-face social interactions. Therefore, it is possible that participants may have placed focus mainly on the content of the intervention that was not associated with the risk of infection. This content was less specific and related to the concept of self-efficacy and its sources in general. This may be the answer why participants in the experimental group reported a higher level of general self-efficacy, and not social self-efficacy beliefs, at Time 2.

It is also worth noting that when verifying the intervention effect on the outcomes, we applied Bonferroni adjustment to control the familywise error rate (FWER), which lowered the threshold for statistical significance from 0.05 to 0.01. As a result, the effect of group assignment on perceived social support at Time 2, with *p* = .017, was not significant. However, in recent years the routine use of Bonferroni correction has been criticized, and there is no formal consensus among researchers on whether this correction should be applied at all [64]. Perneger posits that “the Bonferroni method is concerned with the general null hypothesis (that all null hypotheses are true simultaneously), which is rarely of interest or use to researchers” (p. 1236) [65]. Using this method also increases the likelihood of type II errors [65]. Some authors suggest controlling for false discovery rate (FDR) rather than FWER or simply reporting effect size and/or confidence intervals for effect size instead of Bonferroni correction [66]. To sum up, the conclusions of our study would have been altered if we had used an alternative statistical approach. Without applying Bonferroni adjustment, and as a result, lowering the probability level, the study results would indicate that the participants in the experimental group reported higher perceived social support at post-test (Time 2). Thus, we suggest that the New in Town intervention’s effectiveness in increasing social support requires further investigation. Moreover, in our trial, we used the Bonferroni adjustment for the a priori sample size calculation. With an expected medium effect size (*d* = 0.50), the statistical power of 0.80, and an unadjusted probability level (*p* = .05) power analysis using G*Power [45] would result in a sample size of 128 participants. Thus, without applying Bonferroni correction, the sample size in our study (*N* = 158) could be considered more than sufficient. Before conducting future studies investigating the effectiveness of this and other social self-efficacy interventions, we suggest that researchers should carefully consider if using the Bonferroni adjustment is the best approach, taking into account the trial’s goal and statistical analysis plan [65].

## Limitations, suggestions for future research and conclusion

The present study has limitations. First, the obtained results represent only the short-term effect, and the intervention may have a delayed effect on study outcomes. Future studies investigating the effectiveness of the New in Town and other social self-efficacy interventions could use more time points to examine long-term effects. A second limitation is related to the generalizability of the study results. We collected data from a relatively small sample (*N* = 158). Study participants were relatively young, mostly single or cohabiting, and had short tenure. In addition, most of the participants were women (81.6%). All of the above may limit the generalizability of the results. Future studies need to replicate current trial findings with different samples of internal migrants, including refugees from Ukraine changing their place of residence in Poland. Another limitation is related to deviations from the study protocol. Initially, general self-efficacy was not included as a secondary outcome, and our initial plan was to gather follow-up data also at 8 weeks after baseline. Moreover, the study dropout rate was high (50.6%). Out of the 158 trial participants, 78 completed the post-test assessment, which may indicate that participants did not find the intervention feasible and/or attractive. We also did not collect data on intervention adherence. Future studies will need to further investigate the feasibility of the intervention, gather data on its delivery, and tailor it to prevent dropout [67]. The subsequent studies need to address the topic of user experience of intervention participants, aiming at its enhancement. Finally, the current trial was conducted shortly before the COVID-19 outbreak had reached Poland [68], which may affect its results. The New in Town intervention and its effectiveness require further research attention in safer, post-pandemic times.

To sum up, the results of our study offer preliminary support for the effects of a self-efficacy intervention designed for internal migrants on general self-efficacy beliefs. The New in Town proposes a new, theory-based approach for increasing general self-efficacy and provides an easily accessible support option for internal migrants in Poland. We hope that this trial might pave the way for other studies on interventions targeting migrants and aimed at increasing self-efficacy beliefs—a personal resource that may facilitate psychological adaptation during this challenging life transition [20].

## Supporting information

S1 File CONSORT checklist. (DOC)

S2 File Trial study protocol original Polish version. (DOCX)

S3 File Trial study protocol English translation. (DOCX)
