# Supplementary material for: New in Town—An internet-based self-efficacy intervention for internal migrants: A randomized controlled trial
Source: PLoS One. 2024 Mar 7;19(3):e0299638. doi: 10.1371/journal.pone.0299638 (PMC10919843; doi:10.1371/journal.pone.0299638)
Supplement: S2 File — (DOCX) [file pone.0299638.s002.docx]

­ **Formularz wniosku z Wydziału Psychologii**

|  |  |  |  |
| --- | --- | --- | --- |
| 19.11.2019 |  |  |  |

*Pola zacienione wypełnia Komisja*

**WNIOSEK O DOPUSZCZENIE DO REALIZACJI
BADAŃ EMPIRYCZNYCH Z UDZIAŁEM LUDZI JAKO OSÓB BADANYCH**

| Anna Rogala |
| --- |
| Wnioskodawca: nazwisko i imię |
| *Jak zdobyć przyjaciół w nowym mieście* – efektywność psychologicznej interwencji mobilnej we wzmacnianiu przekonań o własnej skuteczności w nawiązywaniu i utrzymywaniu relacji interpersonalnych |
| Tytuł projektu badawczego |

Do Komisji ds. Etyki Badań Naukowych Wydziału Psychologii

| **Proszę o dopuszczenie do realizacji projektu badawczego przedstawionego poniżej.**  **1. Oświadczenie wnioskodawcy** | | |
| --- | --- | --- |
| - Oświadczam, że wniosek nie był składany i nie jest rozpatrywany w innej komisji etycznej, np. Komisji Senackiej SWPS. - Oświadczam, że znam i rozumiem spoczywające na mnie obowiązki (regulamin Komisji Etycznej, Podstawowe zasady etyczne prowadzenia badań naukowych) i zobowiązuję się do ich przestrzegania.  \| 19.11.2019 \|  \|  \| \| --- \| --- \| --- \| | | |
| Data |  | Podpis wnioskodawcy |

W przypadku wniosku dotyczącego badań wykonywanych w ramach pracy doktorskiej należy podać:

| – |  | – |
| --- | --- | --- |
| Imię i nazwisko promotora |  | Tytuł/stopień promotora |

Oświadczam, że akceptuję projekt badawczy stanowiący przedmiot niniejszego wniosku

| – |  | – |
| --- | --- | --- |
| Data |  | Podpis promotora |

**2. Dane o projekcie badawczym**

| Wniosek składany jest do Komisji po raz pierwszy**:** | ⌧ TAK | | ❑ NIE | Jeśli NIE należy podać datę i przyczynę poprzedniej odmowy w załączniku | |
| --- | --- | --- | --- | --- | --- |
| Państwowe środki na naukę (np. z NCN) ❑  Środku uczelniane (np. z BST) ⌧  Środki własne ❑  Inne ❑ | | 25.11.2019 | | | 25.06.2020 |
| Źródło finansowania | | Data rozpoczęcia badań | | | Data zakończenia badań |

| **Typ badania:**  ❑ badanie kwestionariuszowe  ❑ badanie eksperymentalne  Oświadczam, że badanie prowadzone jest zgodnie z zasadami TREND: ❑ TAK ❑ NIE  TREND: [http://www.cdc.gov/trendstatement/](http://www.cdc.gov/trendstatement/)  ⌧ badanie eksperymentalne z randomizowanymi grupami kontrolnymi (RCT)  Oświadczam, że badanie prowadzone jest zgodnie z zasadami CONSORT: ⌧ TAK ❑ NIE  CONSORT: <http://www.consort-statement.org/>  ❑ badanie jakościowe |
| --- |
| Celem badania jest weryfikacja efektywności psychologicznej interwencji internetowej skierowanej dla migrantów *Jak zdobyć przyjaciół w nowym mieście* we wzmacnianiu przekonań o własnej skuteczności w nawiązywaniu i utrzymywaniu relacji interpersonalnych (ang. *social self-efficacy*). Psychologiczne interwencje internetowe są formą oddziaływania psychologicznego świadczonego online (Sander, Rausch i Baumeister, 2016). Dzięki stronie internetowej użytkownik ma dostęp do treści psychoedukacyjnych oraz interaktywnych ćwiczeń uporządkowanych w postaci modułów tematycznych (Andersson i Titov, 2014). Wyniki badań wskazują, że za pomocą psychologicznych interwencji wykorzystujących nowe technologie można efektywnie wzmacniać poziom przekonań o własnej skuteczności jednostki, czyli jej subiektywnych przekonań dotyczących zdolności do kontroli nad własnym działaniem i zdarzeniami (Cieślak i in., 2016, Rogala i in., 2016).  Przekonania o własnej skuteczności w nawiązywaniu i utrzymywaniu relacji interpersonalnych (ang. *social self-efficacy*) stanowią jeden z ważnych dla człowieka zasobów osobistych (Smith i Betz, 2000, Sherer i in., 1982). Wyniki badań wskazują, że przekonania te są negatywnie skorelowane z lękiem przed bliskością, depresją i poczuciem samotności (Wei, Russell i Zakalik, 2005), zaś pozytywnie skorelowane z osiągnięciami akademickimi i rozwojem zawodowym (Anderson i Betz, 2001). Sugeruje to, że wysoki poziom *social self-efficacy* może być potencjalnie korzystny w adaptacji migrantów. Bazując na tych założeniach opracowano *Jak zdobyć przyjaciół w nowym mieście*, psychologiczną interwencję internetową, której celem jest wzmocnienie *social self-efficacy* dzięki interaktywnym ćwiczeniom bazującym na technikach terapii poznawczo-behawioralnej. Wyniki badań wskazują, że psychologiczne interwencje wykorzystujące nowe technologie korzystające ze wspomnianych technik są skuteczne we wzmacnianiu przekonań o własnej skuteczności (Clarke i in., 2014, Cieślak i in., 2016).  W niniejszym badaniu postawiono hipotezę, że udział w interwencji spowoduje wzrost poziomu *social self-efficacy* (zmienna pierwszorzędowa, ang. *primary outcome*), wzrost satysfakcji z życia i spostrzeganego wsparcia społecznego oraz obniżenie poczucia samotności (zmienne drugorzędowe, ang. *secondary outcomes*), które utrzymają się przez okres 8 tygodni. Dodatkowym celem badania jest zebranie danych na temat użyteczności (ang. *usability*) interwencji oraz analiza powodów, dla których użytkownicy zaprzestają korzystania z interwencji (ang. *dropout analysis*).  **Metoda**  Osoby badane: osoby pełnoletnie, które w ciągu ostatnich 6 miesięcy zmieniły miejscowość zamieszkania i posiadają dostęp do internetu (*N* = 100).  Zmienne: a) *Social self-efficacy* – Self-efficacy Scale (Sherer i in., 1982), b) poczucie samotności – Skala do Pomiaru Poczucia Samotności De Jong Gierveld (de Jong Gierveld i van Tilburg, 1999) c) satysfakcja z życia – Skala Satysfakcji z Życia (Diener i in., 1985), d) spostrzegane wsparcie społeczne – Berlin Social Support Scales (Łuszczyńska, Kowalska, Schwarzer i Schulz, 2002, e) użyteczność – User Experience Questionnaire (Schrepp i in., 2017).  Interwencja: Ćwiczenia zawarte w interwencji zebrane są w **siedmiu modułach** i odnoszą się do źródeł przekonań o własnej skuteczności wyróżnionych przez Bandurę (2004): a) bezpośrednich doświadczeń, b) pośrednich doświadczeń (modelowanie), c) perswazji społecznych, d) stanów somatycznych i emocji.   1. **Zaczynamy!** Psychoedukacja na temat *social self-efficacy*. 2. **Nasze sukcesy.** Psychoedukacja na temat źródeł przekonań o własnej skuteczności; systematyczna ekspozycja uczestników na sytuacje społeczne oraz wzmacnianie ich przekonań o własnej skuteczności dzięki bezpośrednim doświadczeniom. 3. **Doświadczenia innych osób.** Systematyczna ekspozycja na sytuacje społeczne; zwiększanie przekonań o własnej skuteczności poprzez modelowanie. 4. **Dasz sobie radę!** Systematyczna ekspozycja na sytuacje społeczne; zwiększanie przekonań o własnej skuteczności poprzez perswazje społeczne; psychoedukacja na temat wsparcia społecznego. 5. **W zdrowym ciele, zdrowy duch.** Psychoedukacja na temat stanów somatycznych i emocji jako źródła przekonań o własnej skuteczności; psychoedukacja na temat negatywnych myśli; interaktywne ćwiczenia wykorzystujące techniki radzenia sobie z negatywnymi myślami. 6. **Wychodzimy z domu.** Psychoedukacja na temat korzyści z angażowania się w aktywności w czasie wolnym; interaktywne ćwiczenia dotyczące angażowania się w hobby. 7. **Do dzieła!** Psychoedukacja na temat stawiania celów; tworzenie planu nawiązywania i utrzymywania relacji interpersonalnych.   Przebieg badania: Osoby badane zostaną losowo przydzielone do jednej z dwóch grup: Grupa 1 (grupa eksperymentalna; *n* = 50) uzyska dostęp do interwencji przez okres 3 tygodni, Grupa 2 (*n* = 50) stanowić będzie grupę kontrolną (*waitlist control group*). Pomiar poziomu *social self-efficacy*, poczucia samotności, satysfakcji z życia oraz spostrzeganego wsparcia społecznego nastąpi przed przystąpieniem do interwencji (Pomiar 1), po jej zakończeniu (Pomiar 2) oraz po 8 tygodniach od ukończenia interwencji (Pomiar 3). W grupie kontrolnej zachowane zostaną te same odstępy czasowe między pomiarami (odpowiednio 3 i 8 tygodni). Ocena użyteczności interwencji zostanie przeprowadzona po zakończeniu przez osoby badane korzystania z interwencji. |
| Cel i przebieg badania (maksymalnie dwie strony tekstu) |

**3. Dane o wnioskodawcy - osobie odpowiedzialnej za realizację badań**

| Anna | Rogala | | dr | | |
| --- | --- | --- | --- | --- | --- |
| Imię | Nazwisko | | Tytuł/stopień naukowy | | |
| Wydział Psychologii, Uniwersytet SWPS | | | | | |
| Nazwa jednostki (wydział, katedra, zakład) zatrudniającej wnioskodawcę | | | | | |
| Chodakowska 19/31 | Warszawa | | 03-815 | | |
| Ulica nr | Miejscowość | | Kod | | |
| Uniwersytet SWPS, ul. Chodakowska 19/31, 03-815 Warszawa, pokój S114 | | | | | |
| Adres do korespondencji | | | | | |
| anna.rogala@swps.edu.pl | USUNIĘTO DANE OSOBOWE | | - | | |
| E-mail | Tel. | | Fax. | | |
| 4**. Dane o placówkach, w których będą wykonywane badania** | | | | | |
| – | | | | | |
| Nazwa placówki I | | | | | |
| – | | – | | – | |
| Ulica nr | | Miejscowość | | Kod | |

*Dodać kolejne tabele w przypadku występowania następnych placówek*

**5. Ogólna charakterystyka osób badanych**

| Osoby pełnoletnie, które w ciągu ostatnich 6 miesięcy zmieniły miejscowość zamieszkania i posiadają dostęp do internetu (*N* = 100). |
| --- |

*Określić wiek, płeć, podać planowaną liczbę osób badanych i kryteria doboru*

**Czy osoby badane należą do grup wrażliwych**: ❑ TAK ⌧ NIE

(dzieci, osoby w podeszłym wieku, więźniowie, osoby z ograniczeniami poznawczymi i psychicznymi, osoby pozbawione władzy prawnej, osoby z trudnościami w porozumieniu się słownym, pacjenci)

*Jeśli tak, należy uzasadnić, jakie kroki zostaną podjęte celem zapewnienia osobie badanej bezpieczeństwa i minimalizowania dyskomfortu związanego z badaniem.*

**Czy dobór osób badanych zakłada określone kryteria**: ⌧ TAK ❑ NIE

*Jeśli tak, należy uzasadnić, jak zostanie przekazana informacja o zakwalifikowaniu do, ale także o odrzuceniu od udziału w badaniu i jakie kroki zostaną podjęte celem zapewnienia minimalizowania dyskomfortu związanego z badaniem.*

Informacja dla uczestnika będzie zawierać prośbę o udział w badaniu jedynie osób spełniających określone kryteria: 1) pełnoletniość, 2) zmiana miejsca zamieszkania w ciągu ostatnich 6 miesięcy, 3) posiadanie dostępu do internetu. Uczestnicy zostaną również poinformowani, że w przyszłości psychologiczna interwencja internetowa, której efektywność weryfikowana jest w badaniu, będzie powszechnie dostępna w aplikacji mobilnej Beviado (dostępnej również w formie aplikacji webowej).

**Czy udział w badaniu zakłada wgląd w dokumentację medyczną lub psychologiczną osoby badanej:** ❑ TAK ⌧ NIE

*Jeśli tak, należy uzyskać zgodę uczestników i osób odpowiedzialnych za przechowywanie tych informacji oraz przedstawić kroki podjęte celem zapewnienia poufności.*

**Czy udział w badaniu łączy się z utrwaleniem wizerunku lub głosu osoby badanej**:

❑ TAK ⌧ NIE

*Jeśli tak, należy uzyskać zgodę uczestników i przedstawić kroki podjęte celem zapewnienia poufności.*

**Czy udział osoby badanej w badaniu będzie anonimowy**: ❑ TAK ⌧ NIE

*Jeśli nie, należy uzasadnić i przedstawić kroki podjęte celem zapewnienia poufności osobom badanym. W przypadku zbierania adresów e-mailowych od osób badanych, Komisja sugeruje, aby osoby badane zakładały nowe konta e-mailowe dedykowane wyłącznie na cele udziału w danym projekcie badawczym (w takich przypadkach stosowane informacja powinna się znaleźć w świadomej zgodzie).*

Badanie zostanie przeprowadzone z troską o zapewnienie poufności osobom badanym. Osoby badane będą proszone o podanie adresu e-mail, co umożliwi przesłanie im kopii Świadomej zgody na udział w badaniu naukowym, wysłanie zaproszenia do udziału w programie *Jak zdobyć przyjaciół w nowym mieście*, zaproszenia do wypełnienia kwestionariuszy w Pomiarze 2 i Pomiarze 3, a także zidentyfikowanie kwestionariuszy wypełnionych przez tą samą osobę w poszczególnych pomiarach. Adresy e-mail nie będą wykorzystywane w celach innych niż podane powyżej, a osoby badane zostaną zachęcona do zakładania nowych kont e-mailowych dedykowanych wyłącznie na cele udziału w danym projekcie badawczym.

**Czy wszyscy badani zostaną w pełni poinformowani o procedurze badawczej przed rozpoczęciem badania:** ⌧ TAK ❑ NIE

Jeśli nie, odkłamanie nastąpi: ❑ bezpośrednio ❑ po odroczeniu

*Jeśli nie, należy uzasadnić, dlaczego badani nie zostaną w pełni poinformowani o procedurze przed rozpoczęciem badania oraz przedstawić, kiedy i w jakich warunkach pełna informacja zostanie przekazana oraz jakie kroki zostaną podjęte celem minimalizowania dyskomfortu związanego z procedurą.*

**Czy wszyscy badani zostaną poinformowani o zasadach przetwarzania danych ich dotyczących i zasadach przechowywania danych:** ⌧ TAK ❑ NIE

*Jeśli nie, należy uzasadnić. Komisja zwraca uwagę, że zgodnie z obowiązującymi regulacjami prawnymi, każdej osobie badanej przysługuje prawo do informacji o zasadach przetwarzania i przechowywania danych osobowych.*

**Czy wszyscy badani zostaną poinformowani o prawie do wycofania się z udziału w każdym momencie badania, łącznie z prawem do usunięcia wszystkich danych:** ⌧ TAK ❑ NIE

*Jeśli nie, należy uzasadnić. Komisja zwraca uwagę, że możliwość wycofania z udziału w badaniu na każdym z jego etapów, również po zakończeniu, jest podstawowym prawej osób badanych.*

**Czy wszyscy badani zostaną poinformowani o możliwym negatywnym wpływie na ich samopoczucie i skutkach ubocznych:** ❑ TAK ❑ NIE ⌧ NIE DOTYCZY

*Jeśli tak, należy uzasadnić, jakie kroki zostaną podjęte celem zapewnienia osobie badanej bezpieczeństwa i minimalizowania dyskomfortu związanego z badaniem.*

**Czy wszyscy badani zostaną poinformowani o potencjalnych korzyściach z udziału w badaniu:**

⌧ TAK ❑ NIE ❑ NIE DOTYCZY

*Jeśli nie, należy uzasadnić.*

**Czy wszyscy badani otrzymają dane kontaktowe osoby, do której mogą się zwrócić z pytaniami o przebieg badania:** ⌧ TAK ❑ NIE

*Jeśli nie, należy uzasadnić. Komisja zwraca uwagę, że każdej osobie badanej przysługuje prawo do informacji na każdym z etapów badania, również po jego zakończeniu.*

**Czy wszyscy badani udzielą pisemnej zgody na udział w badaniu:** ⌧ TAK ❑ NIE

*Jeśli nie, należy wyjaśnić (zachowanie anonimowości nie wydaje się tu przekonującym argumentem: 1. jest wiele sposób, żeby zachować anonimowość w zgodzie pisemnej, 2. Jeśli badany stoi twarzą w twarz z eksperymentatorem trudno mówić o anonimowości. W przypadku ustnej zgody należy również wyjaśnić wykonalność respektowania prawa osoby badanej do wycofania swoich danych z projektu: kwestia powiązania danych z osobą badaną przy braku danych identyfikujących osobę).  Zgoda ustna jest dopuszczalna przez Kodeks Etyki, Komisja rekomenduje jednak – wszędzie tam, gdzie to możliwe – pisemną zgodę badanych na udział w badaniu.*Osoby badane udzielą zgody na udział w badaniu poprzez wybór odpowiedniej opcji (TAK/NIE) na platformie SurveyMonkey (<https://www.surveymonkey.com>) dedykowanej do przeprowadzania badań online. Połączenie z serwerem platformy jest szyfrowane, co zwiększa bezpieczeństwo przechowywania i transmisji danych.

**6. Procedury**

**Lista stosowanych procedur**

*Należy wymienić bez opisywania*

|  | Procedura | Osoby badane | | Etap badawczy, którego procedura dotyczy: |
| --- | --- | --- | --- | --- |
|  |  | Wszyscy wymienieni w pkt. 5 | Jeśli NIE, określić której podgrupy dotyczy procedura: |  |
| 1 | Ankieta demograficzna | ⌧ TAK ❑ NIE |  | Pomiar 1 |
| 2 | Self-efficacy Scale (Sherer i in., 1982) | ⌧ TAK  ❑ NIE |  | Pomiar 1, Pomiar 2 i Pomiar 3 |
| 3 | Skala do Pomiaru Poczucia Samotności De Jong Gierveld (de Jong Gierveld i van Tilburg, 1999) | ⌧ TAK ❑ NIE |  | Pomiar 1, Pomiar 2 i Pomiar 3 |
| 4 | Skala Satysfakcji z Życia (Diener i in., 1985) | ⌧ TAK ❑ NIE |  | Pomiar 1, Pomiar 2 i Pomiar 3 |
| 5 | Berlin Social Support Scales (Łuszczyńska, Kowalska, Schwarzer i Schulz, 2002) | ⌧ TAK ❑ NIE |  | Pomiar 1, Pomiar 2 i Pomiar 3 |
| 6 | User Experience Questionnaire (Schrepp i in., 2017). | ❑ TAK ⌧ NIE | Grupa eksperymentalna | Pomiar 2 |

**Szczegółowy opis procedur i narzędzi**

***Oświadczenia:***

**Oświadczam, że posiadam prawo do używania wszystkich narzędzi badawczych chronionych prawem autorskim, które wykorzystane będą w przedstawionym projekcie:**  ⌧ TAK ❑ NIE

**Oświadczam, że posiadam prawo do używania wszystkich materiałów (np. grafiki), które wykorzystane będą w przedstawionym projekcie:** ⌧ TAK ❑ NIE

*Należy opisać wszystkie procedury badawcze.*

*Do wniosku należy załączyć niestandardowe narzędzia badawcze (narzędzia Pracowni Testów Psychologicznych, Pracowni Testów Psychologicznych i Pedagogicznych, procedury manualne nie są wymagane). W przypadku badań prowadzonych on-line wskazane jest zamieszczenie linku do ankiety on-line (ewentualnie załączenie wygenerowanego PDF całej ankiety). Narzędzia jako takie nie podlegają ocenie Komisji (Komisja opiniuje protokół badawczy), ale ponieważ stanowią istotną część procedury badawczej, powinny znaleźć się w dokumentacji przedłożonej Komisji.*

| Nazwa procedury: | Ankieta demograficzna | Czas trwania: | 2 minuty |
| --- | --- | --- | --- |
| Opis: | Ankieta demograficzna umożliwiająca scharakteryzowanie badanej próby. Pytania dotyczą wieku, płci, miejsca zamieszkania, wykształcenia, zawodu, stażu pracy. | | |
|  | Czy istnieją zagrożenia dla badanego związane z wykonaniem danej procedury?  ❑ TAK ⌧NIE | | |

| Nazwa procedury: | Self-efficacy Scale (Sherer i in., 1982) | Czas trwania: | 5 minut |
| --- | --- | --- | --- |
| Opis: | Skala do pomiaru przekonań o własnej skuteczności. Składa się z dwóch podskal do pomiaru 1) uogólnionych przekonań o własnej skuteczności (17 pozycji testowych) oraz 2) przekonań o własnej skuteczności w nawiązywaniu i utrzymywaniu relacji z innymi (6 pozycji testowych). Pozostałe pozycje testowe (7) to twierdzenia buforowe. Osoby badane udzielają odpowiedzi na skali 1-5. | | |
|  | Czy istnieją zagrożenia dla badanego związane z wykonaniem danej procedury?  ❑ TAK ⌧NIE | | |

| Nazwa procedury: | Skala do Pomiaru Poczucia Samotności De Jong Gierveld (de Jong Gierveld i van Tilburg, 1999) | Czas trwania: | 3 minuty |
| --- | --- | --- | --- |
| Opis: | Skala do pomiaru poczucia samotności. Składa się z 11 pozycji testowych, z których 6 to zdania negatywnie sformułowane, opisujące brak satysfakcji z kontaktów społecznych, a pozostałe 5, pozytywnie sformułowanych, służy do pomiaru satysfakcji związanej z relacjami interpersonalnymi. Osoby badane udzielają odpowiedzi na skali 1-5. | | |
|  | Czy istnieją zagrożenia dla badanego związane z wykonaniem danej procedury?  ❑ TAK ⌧ NIE | | |

| Nazwa procedury: | Skala Satysfakcji z Życia (Diener i in., 1985) | Czas trwania: | 1 minuta |
| --- | --- | --- | --- |
| Opis: | Skala do pomiaru satysfakcji z życia. Składa się z 4 pozycji testowych. Osoby badane udzielają odpowiedzi na skali 1-7. | | |
|  | Czy istnieją zagrożenia dla badanego związane z wykonaniem danej procedury?  ❑ TAK ⌧ NIE | | |

| Nazwa procedury: | Berlin Social Support Scales (Łuszczyńska, Kowalska, Schwarzer i Schulz, 2002) | Czas trwania: | 4 minuty |
| --- | --- | --- | --- |
| Opis: | W badaniu zostaną wykorzystane trzy skale z baterii testów Berlin Social Support Scales: 1) skala do pomiaru spostrzeganego dostępnego wsparcia (8 pozycji testowych), 2) skala do pomiaru zapotrzebowania na wsparcie (4 pozycje testowe), 3) skala do pomiaru poszukiwania wsparcia (5 pozycji testowych). Osoby badane udzielają odpowiedzi na skali 1-4. | | |
|  | Czy istnieją zagrożenia dla badanego związane z wykonaniem danej procedury?  ❑ TAK ⌧ NIE | | |
| Nazwa procedury: | User Experience Questionnaire (Schrepp i in., 2017) | Czas trwania: | 3 minuty |
| Opis: | Skala do badania satysfakcji z użytkowania interwencji. Zawiera 26 pozycji testowych. Osoby badane udzielają odpowiedzi na skali 1-7. | | |
|  | Czy istnieją zagrożenia dla badanego związane z wykonaniem danej procedury?  ❑ TAK ⌧ NIE | | |

*Dodać kolejne opisy procedur.*

**7. Spis załączników**

|  |  | Liczba załączników: |
| --- | --- | --- |
| 1. | Świadoma zgoda na udział w badaniu naukowym. |  |
| 2. | Narzędzia badawcze. |  |
