# Supplementary material for: New in Town—An internet-based self-efficacy intervention for internal migrants: A randomized controlled trial
Source: PLoS One. 2024 Mar 7;19(3):e0299638. doi: 10.1371/journal.pone.0299638 (PMC10919843; doi:10.1371/journal.pone.0299638)
Supplement: S3 File — (DOCX) [file pone.0299638.s003.docx]

**Application form from the Faculty of Psychology**

|  |  |  |  |
| --- | --- | --- | --- |
| 19.11.2019 |  |  |  |

*Shaded boxes are filled in by the Commission*

**APPLICATION FOR APPROVAL TO CONDUCT**

**EMPIRICAL RESEARCH INVOLVING HUMAN PARTICIPANTS**

| Anna Rogala |
| --- |
| Applicant: surname and name |
| *How to win friends in a new city* – *the effectiveness of psychological mobile intervention in strengthening beliefs about self-efficacy in establishing and maintaining interpersonal relationships* |
| Title of the research project |

To the Research Ethics Committee of the Faculty of Psychology

| **I am asking for permission to conduct the research project presented below.**  **1. Applicant's declaration.** | | |
| --- | --- | --- |
| - I declare that the application has not been submitted and is not being considered by another ethics committee, e.g. the SWPS Senate Committee. - I declare that I know and understand my obligations (regulations of the Ethics Committee, Basic ethical principles of conducting scientific research) and I undertake to comply with them.  \| 19.11.2019 \|  \|  \| \| --- \| --- \| --- \| | | |
| Date |  | The applicant's signature |

In the case of an application concerning research performed as part of a doctoral thesis, the following should be provided:

| – |  | – |
| --- | --- | --- |
| Name and surname of the supervisor |  | The title/degree of the supervisor |

I declare that I accept the research project which is the subject of this application

| – |  | – |
| --- | --- | --- |
| Date |  | Supervisor signature |

**2. Information about the research project**

| The application is submitted to the Commission for the first time: | ⌧ YES | | ❑ NO | If NO, please provide the date and reason for the previous refusal in the attachment | |
| --- | --- | --- | --- | --- | --- |
| State funding for science (e.g. from the National Science Centre) ❑  University funds (e.g. funds for statutory research) ⌧  Researcher's own funds ❑  Other ❑ | | 25.11.2019 | | | 25.06.2020 |
| Source of funding | | Research start date | | | Research completion date |

| **Research type:**  ❑ questionnaire  ❑ experimental study  I declare that the study is conducted in accordance with TREND principles: ❑ YES ❑ NO  TREND: <http://www.cdc.gov/trendstatement/>  ⌧ experimental study with randomized control groups (RCT)  I declare that the study is conducted in accordance with the CONSORT rules: ⌧ YES ❑ NO  CONSORT: <http://www.consort-statement.org/>  ❑ qualitative study |
| --- |
| The study aims to verify the effectiveness of the psychological Internet intervention intended for migrants *How to win friends in a new city* in strengthening beliefs about self-efficacy in establishing and maintaining interpersonal relationships (social self-efficacy). Online psychological interventions are a form of psychological treatment delivered through the Internet (Sander, Rausch, & Baumeister, 2016). Thanks to the website, the user can access psychoeducational content and interactive exercises arranged in thematic modules (Andersson & Titov, 2014). Research results indicate that psychological interventions using new technologies can effectively strengthen the level of beliefs about an individual's self-efficacy, i.e. their subjective beliefs regarding the ability to control their actions and events (Cieślak et al., 2016; Rogala et al., 2016).  Beliefs about self-efficacy in establishing and maintaining interpersonal relationships (social self-efficacy) are one of the essential personal resources for individuals (Smith & Betz, 2000; Sherer et al., 1982). Research results indicate that these beliefs are negatively correlated with fear of intimacy, depression, and a sense of loneliness (Wei, Russell, & Zakalik, 2005) and positively correlated with academic achievement and professional development (Anderson & Betz, 2001). This suggests that a high level of social self-efficacy may be potentially beneficial in the adaptation process of migrants. Based on these assumptions, *How to win friends in a new city* was developed, an online psychological intervention aimed at enhancing social self-efficacy through interactive exercises based on cognitive-behavioral therapy techniques. Research results indicate that psychological interventions using new technologies use these techniques to effectively strengthen self-efficacy beliefs (Clarke et al., 2014; Cieślak et al., 2016).  This study hypothesized that participation in the intervention would increase social self-efficacy (primary outcome variable), increase life satisfaction and perceived social support, and reduce the sense of loneliness (secondary outcome variables), lasting for 8 weeks. Another objective of the study is to collect data on the intervention's usability and analyze why users stop using the intervention (dropout analysis).  **Methods:**  Respondents: adults who have changed residence in the last six months and have access to the Internet (*N* = 100).  Variables: a) Social self-efficacy – Self-efficacy Scale (Sherer et al., 1982), b) sense of loneliness – De Jong Gierveld Loneliness Scale (de Jong Gierveld & van Tilburg, 1999) c) satisfaction with life – Satisfaction With Life Scale (Diener et al., 1985), d) perceived social support – Berlin Social Support Scales (Łuszczyńska, Kowalska, Schwarzer & Schulz, 2002, e) *usability* – User Experience Questionnaire (Schrepp et al., 2017).  Intervention: The exercises included in the intervention are collected in **seven modules** and refer to the headwaters of self-efficacy beliefs distinguished by Bandura (2004): a) direct experiences, b) indirect experiences (modelling), c) social persuasions, and d) somatic states and emotions.  **We are starting!** Psychoeducation on social self-efficacy.  **Our successes.** Psychoeducation on the sources of self-efficacy beliefs, systematic exposure of participants to social situations, and strengthening their self-efficacy beliefs through direct experiences.  **Other people's experiences.** Systematic exposure to social situations; increasing self-efficacy beliefs through modelling.  **You will make it!** Systematic exposure to social situations; increasing self-efficacy beliefs through social persuasions; psychoeducation on social support.  **A sound mind in a sound body.** Psychoeducation on somatic states and emotions as a source of self-efficacy beliefs; psychoeducation about negative thoughts; interactive exercises using techniques for dealing with negative thoughts.  **We are leaving the house.** Psychoeducation on the benefits of engaging in leisure activities; interactive exercises for engaging in hobbies.  **To work!** Psychoeducation on goal setting; creating a plan for establishing and maintaining interpersonal relationships.  The course of the study: The respondents will be randomly assigned to one of two groups: Group 1 (experimental group; *n* = 50) will have access to the intervention for three weeks, and Group 2 (*n* = 50) will be the control group (waitlist control group). The measurement of the level of social self-efficacy, sense of loneliness, satisfaction with life, and perceived social support will take place before starting the intervention (Measurement 1), after its completion (Measurement 2), and after 8 weeks from the end of the intervention (Measurement 3). In the control group, the same time intervals between measurements will be maintained (3 and 8 weeks, respectively). The assessment of the usefulness of the intervention will be carried out after the respondents have finished using the intervention. |
| Purpose and course of the study (maximum two pages of text) |

**3. Data about the applicant - the person responsible for the implementation of the research**

| Anna | Rogala | | dr (Ph.D.) | | |
| --- | --- | --- | --- | --- | --- |
| Name | Surname | | The title/degree | | |
| Faculty of Psychology, SWPS University | | | | | |
| Name of the unit (faculty, department, institute) employing the applicant | | | | | |
| Chodakowska 19/31 | Warszawa | | 03-815 | | |
| Street number | City | | Zip code | | |
| Uniwersytet SWPS, ul. Chodakowska 19/31, 03-815 Warszawa, room S114 | | | | | |
| Postal address | | | | | |
| anna.rogala@swps.edu.pl | PERSONAL INFORMATION REMOVED | | - | | |
| E-mail | Phone number | | Fax. | | |
| 4**. Information about the facilities where the research will be conducted** | | | | | |
| – | | | | | |
| Facility name I | | | | | |
| – | | – | | – | |
| Street number | | City | | Zip code | |

*Add more tables if there are more facilities*

**5. General characteristics of the respondents**

| Adults who have changed their place of residence in the last 6 months and have access to the Internet (*N* = 100). |
| --- |

*Specify age, gender, planned number of respondents, and selection criteria*

**Whether the respondents belong to vulnerable groups**: ❑ YES ⌧ NO

(children, the elderly, prisoners, people with cognitive and mental limitations, people deprived of legal authority, people with difficulties in verbal communication, patients)

*If so, justify what steps will be taken to ensure the safety of the study participant and to minimize the discomfort associated with the study.*

**Does the selection of participants requires certain criteria?**: ⌧ YES ❑ NO

*If so, justify how the information about being selected for, but also about rejection from, participation in the study will be communicated and what steps will be taken to ensure that any discomfort associated with the study is minimized.*

The information for the participant will contain a request to participate in the study only of persons meeting certain criteria: 1) being of age, 2) changing the place of residence in the last 6 months, 3) having access to the Internet. Participants will also be informed that in the future, the online psychological intervention, the effectiveness of which is being verified in the study, will be widely available in the Beviado mobile application (also available as a web application).

**Does participation in the study require access to the medical or psychological records of the examined person:**

❑ YES ⌧ NO

*If so, the consent of the participants and those responsible for storing this information should be obtained and the steps taken to ensure confidentiality should be presented.*

**Does participation in the study involve the recording of the image or voice of the tested person:**

❑ YES ⌧ NO

*If so, consent from participants should be obtained and steps taken to ensure confidentiality should be provided.*

**Will be the participation in the study anonymous**: ❑ YES ⌧ NO

*If not, the steps taken to ensure the confidentiality of the participant should be justified and presented. In the case of collecting e-mail addresses from the participants, the Commission suggests that the participants set up new e-mail accounts dedicated solely for the purposes of participation in a given research project (in such cases, the information used should be included in the informed consent).*

The study will be conducted with care to ensure the confidentiality of the respondents. Participants will be asked to provide their e-mail address, which will allow them to be sent a copy of the Informed Consent to Participate in the Research Study, an invitation to participate in the *How to win friends in a new city* program, an invitation to complete the questionnaires in Measurement 2 and Measurement 3, as well as identifying questionnaires completed by the same person in individual measurements. E-mail addresses will not be used for purposes other than those listed above, and the respondents will be encouraged to set up new e-mail accounts dedicated solely to the purpose of participating in a given research project.

**Will all respondents be fully informed about the research procedure before the start of the research:** ⌧ YES ❑ NO

If not, the information about the research procedure will be provided ❑ immediately after the research participation ❑ some period of time after the research participation

*If not, please provide the justification for why respondents do not receive a complete information about the study procedure prior to research participation and please declare when and under which circumstances the complete information about the study procedure will be provided to respondents, and what means will be used in order to minimize discomfort associated with the study participation.*

**Will all respondents be informed about the data processing and data storage policies:** ⌧ YES ❑ NO

*If not, please justify. The Commission points out that in line with legal regulations, each respondent has the right to be informed about the rules of data processing and storing.*

**Will all respondents be informed of the right to withdraw from participation at any time in the study, including the right to delete all data:** ⌧ YES ❑ NO

*If not, please justify. The Commission points out that the possibility of withdrawing from participation in the study at each of its stages, also after completion, is the elementary right of the participant.*

**Will all respondents be informed about the potential negative impact on their well-being and about the side effects of study participation:** ❑ YES ❑ NO ⌧ NOT APPLICABLE

*If so, justify what steps should be taken to ensure the respondent's safety and minimize the discomfort associated with the study participation.*

**Will all respondents be informed about the potential benefits of participating in the study:**

⌧ YES ❑ NO ❑ NOT APPLICABLE

*If not, please justify.*

**Will all participants receive contact details of a person they can contact with questions about the course of the study:** ⌧ YES ❑ NO

*If not, justify. The Commission points out that each examined person has the right to information at each stage of the research, also after its completion.*

**Will all respondents provide written consent to participate in the study:** ⌧ YES ❑ NO

*If not, please explain (maintaining anonymity does not seem to be a convincing argument here: 1. there are many ways to maintain anonymity in written consent, 2. If the respondent is face-to-face with the experimenter, it is difficult to talk about anonymity. In the case of verbal consent, also explain the feasibility of respecting the right of the test person to withdraw their data from the project: the question of linking the data to the respondent in the absence of data identifying the person). Verbal consent is allowed by the Code of Ethics, however, the Committee recommends - wherever possible - written consent of the respondents to participate in the study.*

Respondents consent to participate in the study by selecting the appropriate option (YES/NO) on the SurveyMonkey platform (https://www.surveymonkey.com) dedicated to conducting online surveys. The connection to the platform server is encrypted, which increases the security of data storage and transmission.

**6. Procedure**

**List of procedures**

*Please provide the list of the procedures without a detailed description*

|  | Procedure | Respondents | | The research stage to which the procedure applies: |
| --- | --- | --- | --- | --- |
|  |  | All those listed in point 5 | If NO, specify which subgroup the procedure applies to: |  |
| 1 | Demographic survey | ⌧ YES ❑ NO |  | Measurement 1 |
| 2 | Self-efficacy Scale (Sherer et al.., 1982) | ⌧ YES  ❑ NO |  | Measurement 1, Measurement 2, and Measurement 3 |
| 3 | Loneliness Scale De Jong Gierveld (de Jong Gierveld & van Tilburg, 1999) | ⌧ YES ❑ NO |  | Measurement 1, Measurement 2, and Measurement 3 |
| 4 | Satisfaction with Life Scale (Diener et al., 1985) | ⌧ YES ❑ NO |  | Measurement 1, Measurement 2, and Measurement 3 |
| 5 | Berlin Social Support Scales (Łuszczyńska, Kowalska, Schwarzer & Schulz, 2002) | ⌧ YES ❑ NO |  | Measurement 1, Measurement 2, and Measurement 3 |
| 6 | User Experience Questionnaire (Schrepp et al.., 2017) | ❑ YES ⌧ NO | Experimental group | Measurement 2 |

**A detailed description of the procedure and tools**

***Statements:***

**I declare that I have the right to use all research tools protected by copyright that will be used in the presented project:**

⌧ YES ❑ NO

**I declare that I have the right to use all materials (e.g. graphics) that will be used in the presented project:**

⌧ YES ❑ NO

*All test procedures should be described.*

*Non-standard research tools should be attached to the application (tools of the Psychological Tests Laboratory, Psychological, and Pedagogical Tests Laboratory, manual procedures are not required). In the case of research conducted online, it is advisable to include a link to the online survey (or attach a generated PDF of the entire survey). The tools as such are not subject to evaluation by the Commission (the Commission gives its opinion on the research protocol), but since they are an essential part of the research procedure, they should be included in the documentation submitted to the Commission.*

| Procedure name: | Demographic survey | Duration: | 2 minutes |
| --- | --- | --- | --- |
| Description: | Demographic survey enabling the characterization of the tested sample. The questions concern age, gender, place of residence, education, profession, work experience. | | |
|  | Are there any risks to the respondent related to the procedure?  ❑ YES ⌧NO | | |

| Procedure name: | Self-efficacy Scale (Sherer et al., 1982) | Duration: | 5 minutes |
| --- | --- | --- | --- |
| Description: | A scale for measuring self-efficacy beliefs. It consists of two subscales to measure 1) generalized self-efficacy beliefs (17 test items) and 2) self-efficacy beliefs in establishing and maintaining relationships with others (6 test items). The remaining test items (7) are buffer statements. Respondents give answers on a scale of 1-5. | | |
|  | Are there any risks to the respondent related to the procedure?  ❑ YES ⌧NO | | |

| Procedure name: | Loneliness Scale De Jong Gierveld (de Jong Gierveld & van Tilburg, 1999) | Duration: | 3 minutes |
| --- | --- | --- | --- |
| Description: | A scale for measuring the sense of loneliness. It consists of 11 test items, of which 6 are negatively formulated sentences, describing the lack of satisfaction with social contacts, and the remaining 5, positively formulated, are used to measure satisfaction related to interpersonal relationships. Respondents give answers on a scale of 1-5. | | |
|  | Are there any risks to the respondent related to the procedure?  ❑ YES ⌧ NO | | |

| Procedure name: | Satisfaction with Life Scale (Diener et al., 1985) | Duration: | 1 minute |
| --- | --- | --- | --- |
| Description: | A scale for measuring satisfaction with life. It consists of 4 test positions. Respondents give answers on a scale of 1-7. | | |
|  | Are there any risks to the respondent related to the procedure?  ❑ YES ⌧ NO | | |

| Procedure name: | Berlin Social Support Scales (Łuszczyńska, Kowalska, Schwarzer & Schulz, 2002) | Duration: | 4 minutes |
| --- | --- | --- | --- |
| Description: | Three scales from the Berlin Social Support Scales test battery will be used in the study: 1) a scale for measuring the perceived available support (8 test items), 2) a scale for measuring the need for support (4 test items), 3) a scale for measuring support seeking ( 5 test items). Respondents answer on a scale of 1-4. | | |
|  | Are there any risks to the respondent related to the procedure?  ❑ YES ⌧ NO | | |
| Procedure name: | User Experience Questionnaire (Schrepp et al., 2017) | Duration: | 3 minutes |
| Opis: | Scale for examining satisfaction with the use of internet intervention. Contains 26 test items. Respondents give answers on a scale of 1-7. | | |
|  | Are there any risks to the respondent related to the procedure?  ❑ YES ⌧ NO | | |

*Add more procedure descriptions.*

**7. List of attachments**

|  |  | Number of attachments: |
| --- | --- | --- |
| 1. | Informed consent form to participate in a research study. |  |
| 2. | Research tools. |  |
